# Comparison of efficacy and safety between aspirin and oral anticoagulants for venous thromboembolism prophylaxis after major orthopaedic surgery: a meta-analysis of randomized clinical trials

**DOI:** 10.3389/fphar.2023.1326224

**Published:** 2024-01-08

**Authors:** Xingyue Zheng, Li Nong, Yujie Song, Lizhu Han, Yuan Zhang, Qinan Yin, Yuan Bian

**Affiliations:** ^1^ Personalized Drug Therapy Key Laboratory of Sichuan Province, School of Medicine, University of Electronic Science and Technology of China, Chengdu, China; ^2^ Department of Pharmacy, People’s Hospital of Guangxi Zhuang Autonomous Region, Nanning, China; ^3^ Department of Pharmacy, Sichuan Academy of Medical Sciences and Sichuan Provincial People’s Hospital, Chengdu, China

**Keywords:** major orthopaedic surgery, aspirin, oral anticoagulation, venous thromboembolism prophylaxis, systematic review

## Abstract

**Background:** venous thromboembolism (VTE) is one of the most common complications after major orthopaedic surgery. Recent studies have suggested that aspirin may also be effective in preventing VTE, but it is still controversial whether it can be routinely used.

**Objectives:** To compare the efficacy and safety of aspirin against oral anticoagulants in the prevention of VTE following total hip arthroplasty (THA), total knee arthroplasty (TKA) or hip fracture surgery (HFS).

**Methods:** Relevant publications have been obtained using electronic search databases such as PubMed, Embase, Web of Science, Cochrane Library, and Clinical Trials. gov. from inception to 20 July 2023. Only RCTs evaluating the efficacy and safety of aspirin compared with oral anticoagulants undergoing major orthopaedic surgery were included in the meta-analysis. The primary outcome reported was any VTE event (including deep vein thrombosis (DVT) and pulmonary embolism (PE)). Secondary outcomes included mortality, major bleeding (including gastrointestinal bleed, cerebrovascular hemorrhage, or any bleeding requiring a return to the theater), minor bleeding (ecchymosis, epistaxis, hematuria), and wound complications. The risk of bias for all included studies was assessed according to the Cochrane Collaboration’s tool.

**Results:** After screening 974 studies, 12 randomized clinical trials (RCTs) were included, involving 5,088 participants, including 2,540 participants in aspirin, 2,205 participants in rivaroxaban, and 323 participants in warfarin. Aspirin was found to be less effective than oral anticoagulants in thromboprophylaxis after major orthopedic surgery (RR = 1.206, 95% CI 1.053–1.383). After subgroup analysis according to the type of oral anticoagulant, the results showed that aspirin was similar to rivaroxaban and inferior to warfarin. Considering that the studies in the warfarin group were all conducted before 2000, our results need to be further confirmed. In addition, the aspirin group had a higher risk of VTE than the control group in other subgroups, including a follow-up time of ≤3 months, type of procedure as TKA, high-dose aspirin (≥650 mg qd), and no combined use of mechanical prophylaxis. In terms of safety events, aspirin did not show significant differences in major bleeding (RR = 0.952, 95% CI 0.499–1.815), all-cause mortality (RR = 1.208, 95% CI 0.459–3.177), and wound-related events (RR = 0.618, 95% CI 0.333–1.145) compared with oral anticoagulants, and aspirin was associated with a reduction in the risk of minor bleeding (RR = 0.685, 95% CI 0.552–0.850) events and total bleeding (RR = 0.726, 95% CI 0.590–0.892).

**Conclusion:** Aspirin reduces bleeding risk after major orthopedic surgery compared with oral anticoagulants, but may sacrifice VTE prevention to some extent. Updated evidence is needed to analyze the thromboprophylaxis effects of aspirin in patients undergoing major orthopedic surgery.

**Systematic Review Registration:** https://www.crd.york.ac.uk/prospero/display_record.php?RecordID=463481, identifier CRD42023463481.

## 1 Introduction

Patients undergoing major orthopaedic surgery, such as total knee arthroplasty (TKA) and total hip arthroplasty (THA), and hip fracture surgery (HFS), are at elevated risk of venous thromboembolism (VTE), including deep vein thrombosis (DVT) and pulmonary embolism (PE) (Piovella et al., 2005; Learmonth et al., 2007; Memtsoudis et al., 2012). Without prevention of thrombosis, post-operative thrombosis of THA and TKA can reach as high as 42%–57% and 40%–80% (Piovella et al., 2005; Geerts et al., 2008; Fisher, 2011), respectively, and up to 2% for fatal PE (Geerts et al., 2004; Howie et al., 2005; Kinov et al., 2014).

Current guidelines recommend prophylaxis should be given for a minimum of 10–14 days in patients undergoing major orthopaedic surgery (Chinese Orthopaedic Association, 2010; Falck-Ytter et al., 2012; Anderson et al., 2019; Gee, 2019). The common pharmacological agents for VTE prophylaxis include direct oral anticoagulants (DOACs) like rivaroxaban, heparin-based agents, vitamin K antagonists, and aspirin. Although there is clinical evidence for the use of these drugs, studies have shown differences in bleeding risk with their use, including major bleeding and increased readmission rates (Zou et al., 2014; Feng et al., 2015; Messerschmidt and Friedman, 2015). According to the Asia-Pacific venous thromboembolism consensus in knee and hip arthroplasty and hip fracture surgery announced in 2021, among all available pharmacological agents for VTE prophylaxis, aspirin is the most appropriate and more cost-effective prophylactic agent than other agents for Asians undergoing elective knee and hip arthroplasty with standard VTE risk, while for patients with elevated VTE risk, DOACs or LMWH are the most appropriate agents (Thiengwittayaporn et al., 2021). However, which pharmacological agent is the most appropriate for Asians undergoing hip fracture surgery still lacks enough supporting evidence. Guideline consensus differs in the prophylaxis of VTE for major orthopaedic surgery.

Randomized controlled trials have been published to evaluate the clinical efficacy and safety of aspirin for the prevention of VTE after THA, TKA, or HFS compared to oral anticoagulants, and the current guideline consensus is still to recommend anticoagulation, but aspirin is also recommended (Jenny et al., 2018; Anderson et al., 2019; Gee, 2019). We conducted a meta-analysis of randomized clinical trials (RCTs) comparing aspirin oral anticoagulants in patients after THA, TKA, or HFS.

## 2 Materials and methods

We performed a meta-analysis of RCTs according to the guidelines in Preferred Reporting Items for Systematic Reviews and Meta-Analyses (PRISMA).

### 2.1 Search strategy

A comprehensive search was conducted independently by two individuals using PubMed, Embase, Web of Science, Cochrane Library, and ClinicalTrials.gov databases, covering the period from the inception of the databases until 20 July 2023. The computer-based searches combined terms and combinations of keywords related to the participants (e.g., hip arthroplasty, knee arthroplasty, hip replacement, knee replacement, or hip fractures surgery), drug intervention (e.g., aspirin), comparators (e.g., anticoagulants, warfarin, rivaroxaban, dabigatran, apixaban, or edoxaban), and outcomes (e.g., venous thromboembolism, pulmonary embolism, cerebral hemorrhage, or gastrointestinal hemorrhage). The reference list of all the retrieved articles underwent a manual examination to uncover additional potentially relevant studies. This study was registered in PROSPERO(CRD42023463481).

### 2.2 Inclusion and exclusion criteria

The search was limited to humans. Case reports of fewer than five neonates and conference abstracts were excluded.

The inclusion criteria encompassed the following: Studies were included if they met the following criteria: 1) Patient underwent major orthopedic surgery including TKA, THA, and HFS; 2) only randomized controlled trials were included; 3) treatment had to involve aspirin and oral anticoagulants, and oral anticoagulants included DOACs and warfarin; 4) the primary study outcome was reported, which was the occurrence of any VTE (including DVT and/or PE) after the procedure, regardless of whether the event was asymptomatic or asymptomatic. Secondary outcomes reported included mortality, major bleeding events (including gastrointestinal bleeding and cerebrovascular bleeding), other bleeding complications, and wound-related problems (e.g., hematomas and infections). There was no limit to the length of follow-up for inclusion in the study.

The exclusion criteria were defined as follows: Those articles that met the following criteria were excluded: 1) articles displaying inconsistent research content; 2) the type of article was review articles, conference abstracts, studies involving animals, case reports or study protocols; 3) articles not in English or Chinese.

### 2.3 Data extraction and risk of bias assessment

Two reviewers (Z.X.Y and L.N) independently screened the titles and abstracts and reviewed the full text, and articles that met the criteria were included by reviewing the full text, and then study characteristics, baseline characteristics, and prespecified efficacy and safety outcomes were extracted. Selected studies were scored based on The Cochrane Risk of Bias criteria (Cochrane Collaboration, 2011), which includes seven types of risk of bias assessment: random sequence generation, allocation concealment, blinding of participants and personnel, blinding of outcome assessment, incomplete outcome data, selective reporting, and other bias.

### 2.4 Statistical methods

We calculated the relative risk (RR) and 95% confidence interval (CI) for each study. Heterogeneity was assessed using the I^2^ statistic. If I^2^ ≤ 50% and *p* ≥ 0.1, indicating that there was no heterogeneity between study outcomes, a fixed-effects model was used. If I^2^ > 50% and *p* < 0.1 indicated that there was heterogeneity among the study outcomes, a random effects simulation was used to analyze the source of heterogeneity (Higgins et al., 2003). If the event rate was zero, we corrected for half-integer continuity for all four cells (Friedrich et al., 2007). Publication bias was assessed using the Egger test and Begg’s test. If there was any publication bias, the trim and fill methods were used to adjust for publication bias (Duval and Tweedie, 2000). We performed sensitivity analyses for each outcome by excluding one study at a time to assess the stability of the meta-analysis results. To assess the impact of potential factors on the results of the meta-analysis of the primary outcome, we conducted several subgroup analyses based on study-level characteristics. All statistical analyses were performed using STATA software (version 15.0), and *p* < 0.05 indicates a statistically significant difference.

## 3 Result

### 3.1 Study identification and characteristics

**FIGURE 1:**
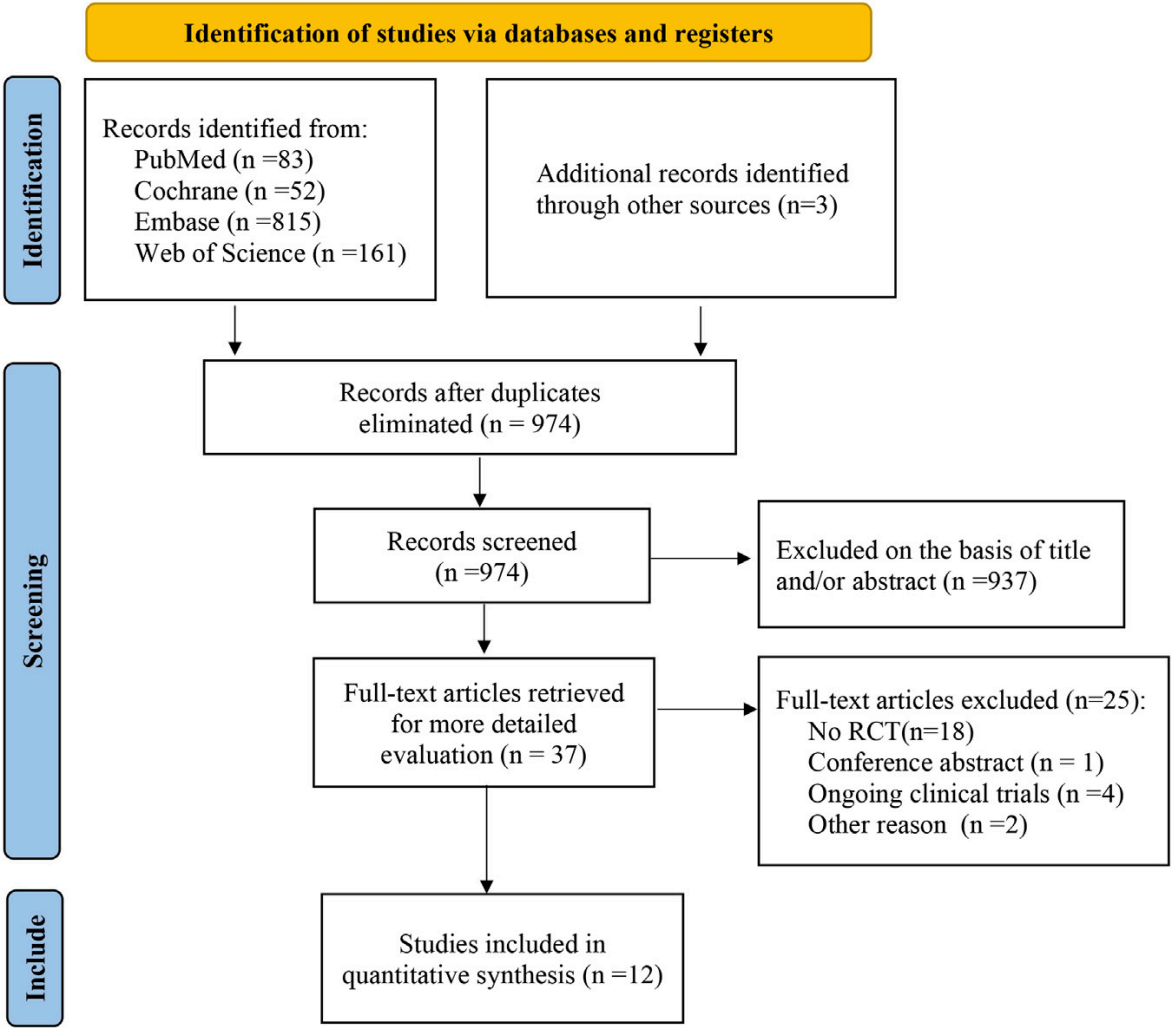
Flow chart of the selected study.

As of July 2020, a total of 974 potentially relevant citations were identified (Figure 1), 937 irrelevant citations were excluded based on title and/or abstract. In addition, one conference abstract, 18 non-RCT studies, four ongoing clinical trials, and three articles were excluded for other reasons. Finally, 12 randomized controlled trials were included. A flowchart for retrieval and inclusion was created based on the PRISMA guidelines (Figure 1). The PRISMA checklist was provided in Supplementary Table S1. The characteristics of the included studies are listed in Table 1. The 12 randomized controlled trials included 5,088 patients, of which 6 studies compared aspirin with rivaroxaban, 4 studies compared aspirin with warfarin, and 2 studies compared aspirin and low-molecular heparin bridged to rivaroxaban. All 12 studies reported VTE outcomes, 11 studies reported fatal events, 10 studies reported major bleeding and minor bleeding, and 8 studies reported wound-related issues.

**TABLE 1:** Summary characteristics of the included 12 randomized clinical trials.

Study	Participants	Mean age (years)		Male (%)		Surgery	Mechanical prophylaxis	Dosage and duration		Time follow up	DVT diagnostic methods
		Aspirin	Comparator	Aspirin	Comparator			Aspirin	Comparator		
Anderson et al. (2018)	3424	62.9 ± 10.1	62.7 ± 10.1	47.1	48.5	THA:1804; TKA:1620	NS	Rivaroxaban 10 mg qd for 5 days, then switched to aspirin 81 mg for 9 days after TKA or 30 days after THA	Rivaroxaban 10 mg for 14 days after TKA or 35 days after THA	90 days	Compression ultrasonography; ventilation–perfusion scan or CTPA
Colleoni et al. (2018)	32	71.21 ± 6.35	67.11 ± 7.65	7.10	22.20	TKA	NS	Aspirin 300 mg qd for 14 days	Rivaroxaban 10 mg qd for 14 days	4 weeks	Doppler ultrasonography
Hongnaparak et al. (2022)	40	68.15 ± 7.43	70.5 ± 7.25	35	15	TKA	NS	Aspirin 300 mg qd for 14 days	Rivaroxaban 10 mg qd for 14 days	14 days	Duplex ultrasonography and SCT
Huang et al. (2019)	390	69.4 ± 17.4	67.8 ± 16.9	50	47.7	HFS	NS	Enoxaparin for 5 days followed by aspirin 100 mg qd for 16 days	Enoxaparin for 5 days followed by rivaroxaban 10 mg qd for 16 days	90 days	Lower extremity venous ultrasound and probe compression; CTPA
Jiang et al. (2014)	120	65.1 ± 7.5	63.8 ± 6.7	8.33	6.67	TKA	TED stockings and IPC	Aspirin 100 mg qd for 14 days	LMWH (5 000 IU/day) for 5 days, followed by rivaroxaban 10 mg qd for 9d	6 weeks	Duplex ultrasonography
Lotke et al. (1996)	312	66.4	67.1	41.25	36.67	THA: 133 TKA: 179	NS	Aspirin 325 mg bid for 6 weeks	warfarin (dosage adjusted for prothrombin time) for 6 weeks	6 months	Ventilation perfusion scans and venograms
Powers et al. (1989)	131	28.8	35.4	73.0	74.5	HFS	NS	Aspirin 650 mg bid for 21 days	warfarin (dosage adjusted for prothrombin time) for 21 days	3 months	Iodine 125-fibrinogen leg scanning and impedance plethysmography, with subsequent venography
Ren et al. (2021)	80	38.3	30.6	54.5	50.0	THA	Pneumatic compression for two groups	100 mg aspirin bid for 5 weeks	10 mg rivaroxaban qd for 5 weeks	3 months	Doppler ultrasonography
Salzman et al. (1971)	86	51	48	NS	NS	THA	NS	Aspirin, 600 mg bid, for 21–35 days	Warfarin (Dosage adjusted for prothrombin time) for 21–35 days	NS	Ventilation perfusion scans and venograms
Woolson and Watt (1991)	141	62.3	67.6	50	45	THA	IPC plus elastic stockings for two groups	Aspirin 650 mg bid, duration: NS	Warfarin (Dosage adjusted for prothrombin time), duration: NS	3 months	Venography or venous ultrasonography
Zou et al. (2014)	212	62.7	63.5	25.45	31.37	TKA	NS	Aspirin 100 mg qd for 14 days	Rivaroxaban 10 mg qd for 14 days	4 weeks	Doppler ultrasonography
Zhou et al. (2023)	120	66.4 ± 7.6	64.8 ± 7.2	43.33	45	TKA	NS	Aspirin 100 mg qd for 30 days	Rivaroxaban 10 mg qd for 30 days	3.02 ± 0.09 months	Lower extremity venous ultrasound; CTPA

NS, not stated; TKA, total knee arthroplasty; THA, total hip arthroplasty; HFS, hip fracture surgery; TED, thromboembolic deterrent; IPC, intermittent pneumatic compression; CTPA, computed tomographic pulmonary angiography; SCT, spiral computed tomography.

### 3.2 Risk of bias

**FIGURE 2:**
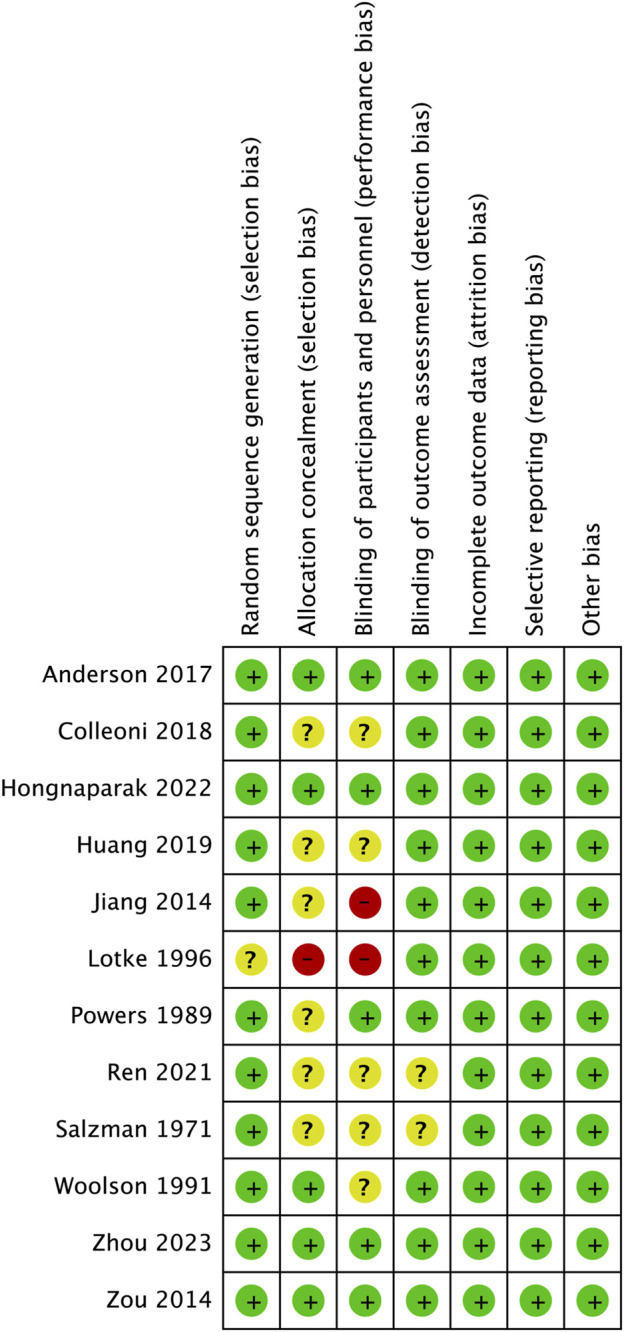
Risk of bias graph.

**FIGURE 3:**
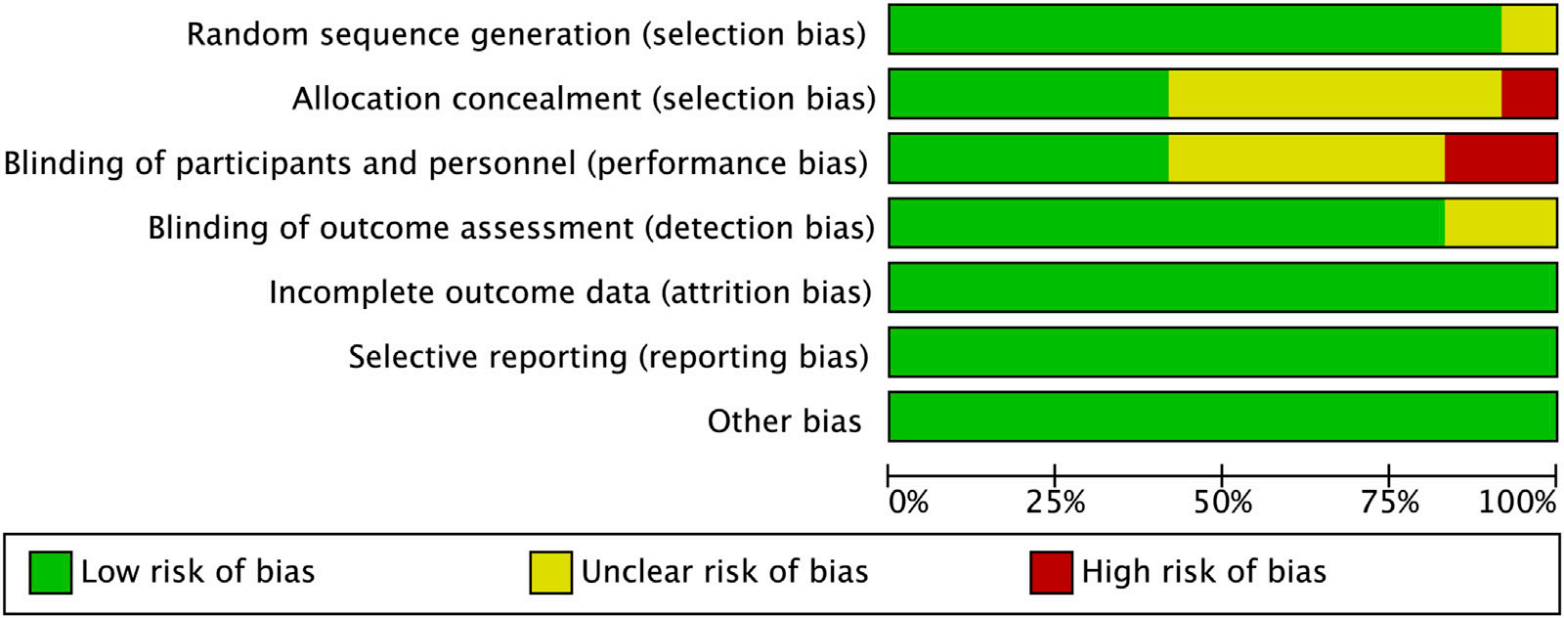
Risk of bias summary.

The risk of bias for each study assessed by RevMan 5.3 software is shown in Figures 2, 3. The main bias was allocation concealment and blinding of participants and personnel and personnel and personnel. Allocation blinding was not performed because the control group required subcutaneous injections of LMWH in the study by Jiang et al. (2014). The study of Lotke et al. (1996) was not blinded for the rest of the study except for the diagnostic phase, for which no reason was given. Egger’s test showed no significant publication bias in the results of most studies, except all-cause mortality (*p* = 0.035). The results of the egger test were listed in Supplementary Table S2.

### 3.3 Sensitivity analyses

The overall results failed to identify any individual trials that had a significant impact on the results (Supplementary Tables S3–S8).

### 3.4 Primary outcome

**FIGURE 4:**
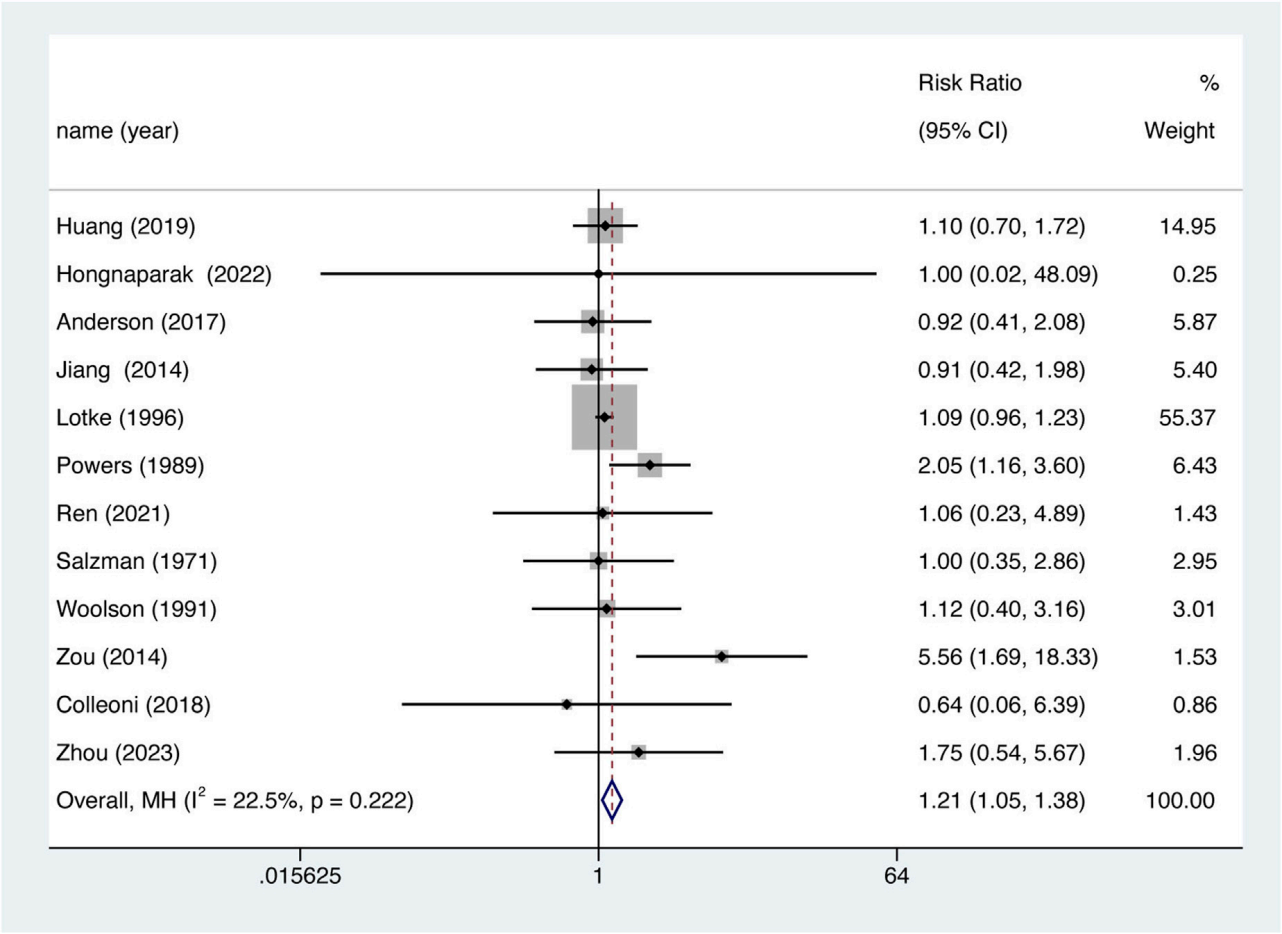
Comparison of the efficacy of aspirin versus oral anticoagulant in VTE.

The occurrence of VTE was recorded as an outcome in every study. 12 studies were included in the analysis, Overall, aspirin was associated with lower VTE prevention efficiency. (Figure 4, RR = 1.206, 95% CI 1.053–1.383, *p* = 0.007), and there was no significant heterogeneity between studies (I^2^ = 22.5%, df = 11, *p* = 0.222). The results of the meta-analysis including 95% CI as well as *p* values for all clinical outcomes are summarized in Supplementary Table S9.

### 3.5 All-cause mortality

**FIGURE 5:**
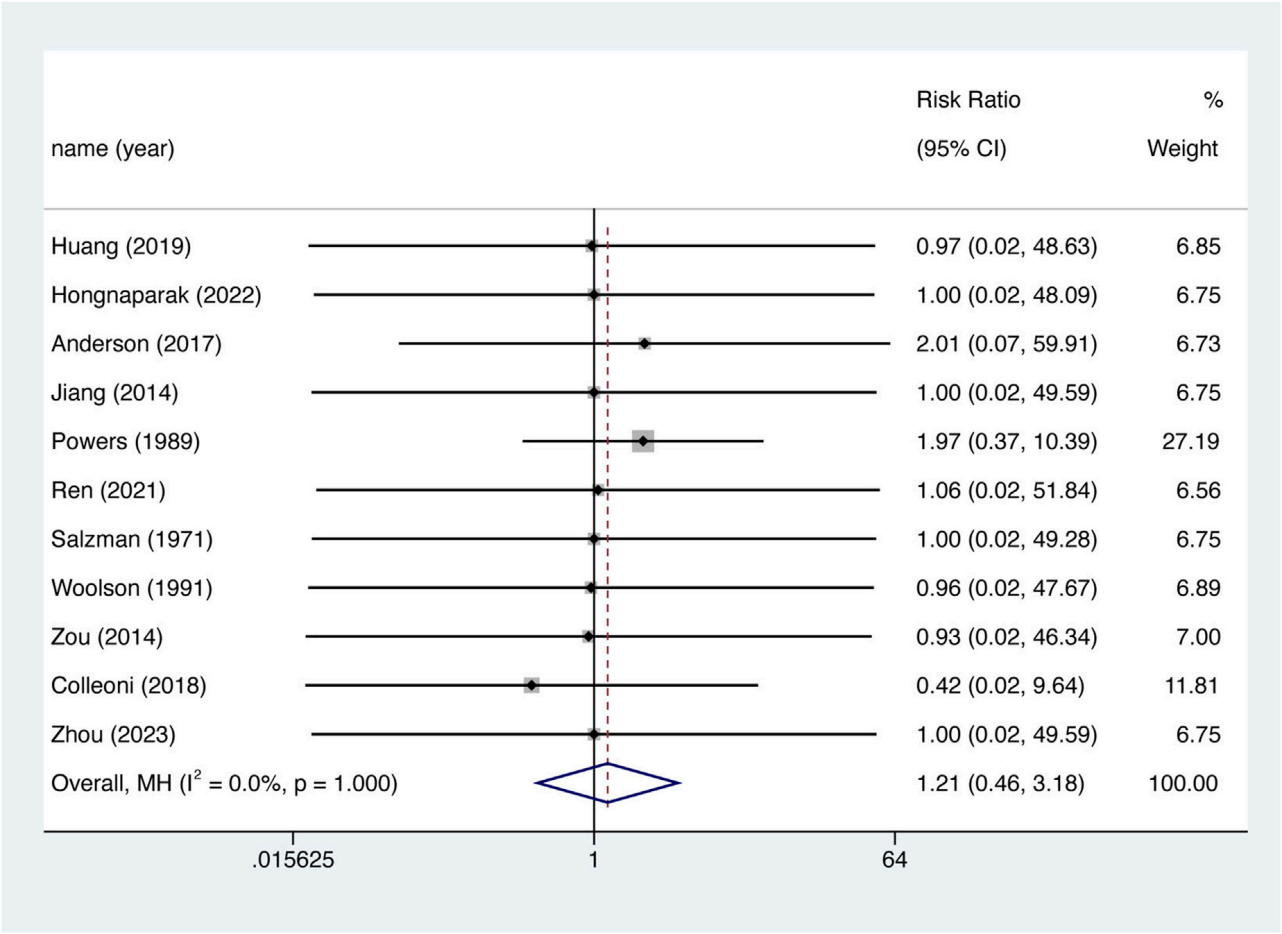
Death events between aspirin and oral anticoagulant.

11 studies reported all-cause mortality rates. However, the incidence of fatal events was low, with only three studies reporting such events (Powers et al., 1989; Anderson et al., 2018; Colleoni et al., 2018). Corrections were made using half-integer continuity, and 11 studies were finally included. There was no significant difference in the risk of death between the two groups (Figure 5. RR = 1.208, 95% CI 0.459–3.177, *p* = 0.702), nor was there significant between-study heterogeneity (I^2^ = 0%, df = 10, *p* = 1.000). The Egger test showed publication bias in the results for all-cause mortality (*p* = 0.035). After the imputation of potentially missing studies using trim-and-fill, the pooled all-cause mortality RR results were similar (RR = 1.217, 95% CI 0.464–3.192, *p* = 0.689).

### 3.6 Hemorrhage events

**FIGURE 6:**
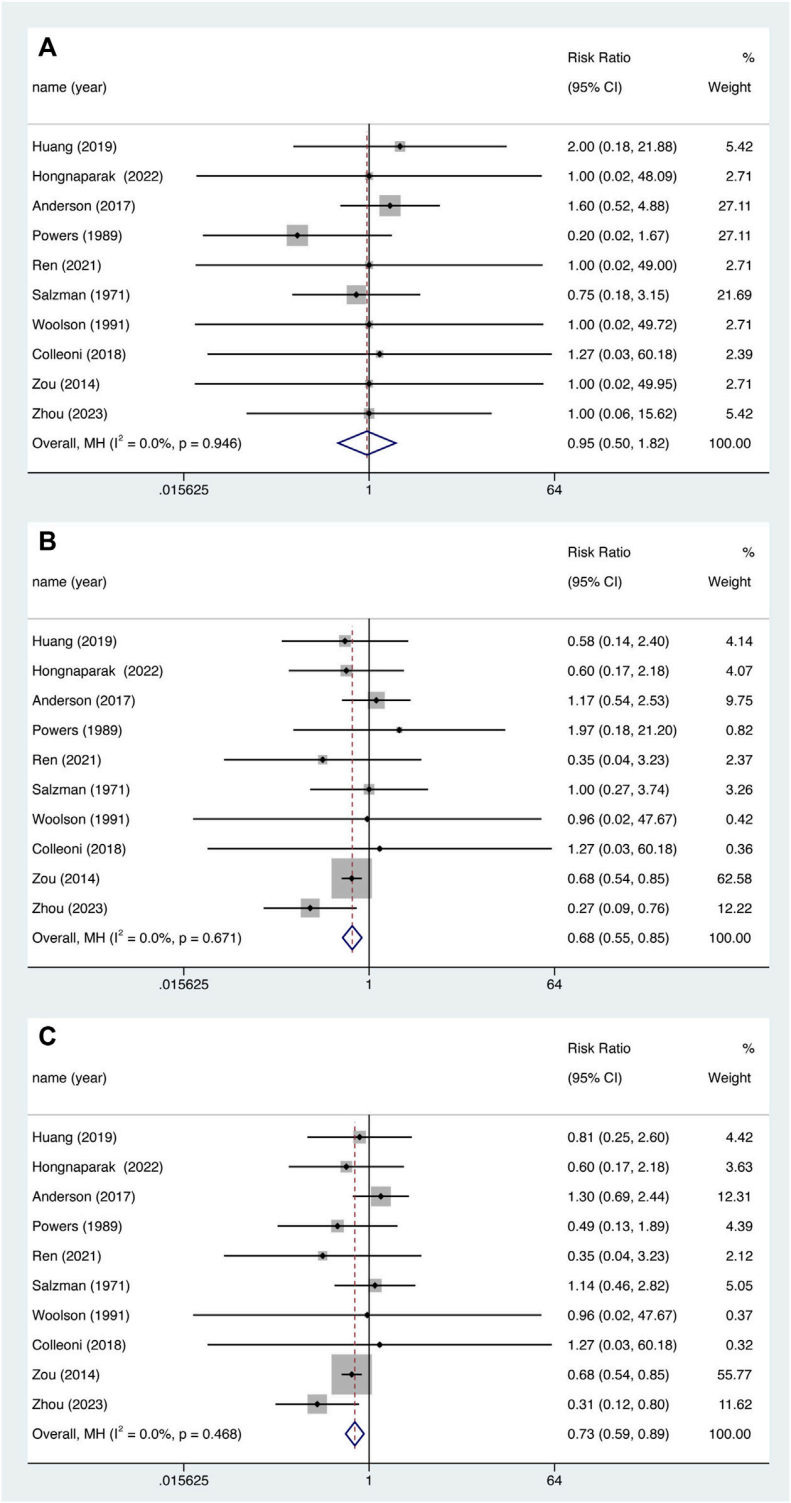
Major bleeding **(A)**, minor bleeding **(B)** and total bleeding events **(C)** between aspirin and oral anticoagulant.

Nine studies reported major bleeding events and minor bleeding events. There was no statistically significant data shown for major bleeding (Figure 6A. RR = 0.952, 95% CI 0.499–1.815, *p* = 0.882), Instead, aspirin was associated with lower risk of minor bleeding (Figure 6B. RR = 0.685, 95% CI 0.552–0.850, *p* = 0.001) and total bleeding (Figure 6C. RR = 0.726, 95% CI 0.590–0.892, *p* = 0.002), and there was no significant heterogeneity between studies (All the I^2^ were 0.0%).

### 3.7 Wound-related issues

**FIGURE 7:**
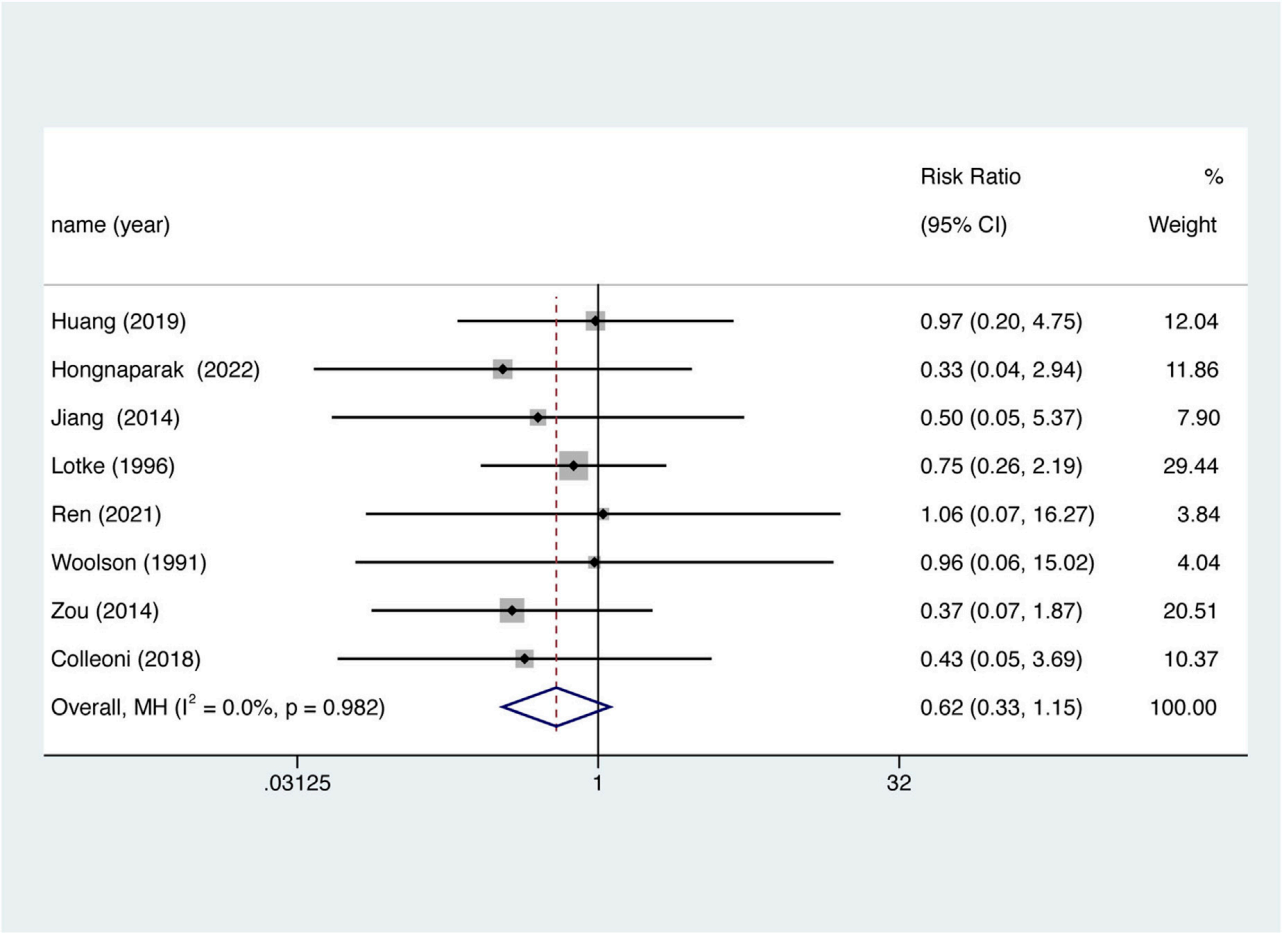
Wound-related issues between aspirin and oral anticoagulant.

Eight studies reported wound-related issues and were included. There was no significant difference in the risk of wound-related issues between the two groups (Figure 7. RR = 0.618, 95% CI 0.333–1.145, *p* = 0.126), nor was there significant between-study heterogeneity (I^2^ = 0%, df = 7, *p* = 0.982).

### 3.8 Subgroup

**FIGURE 8:**
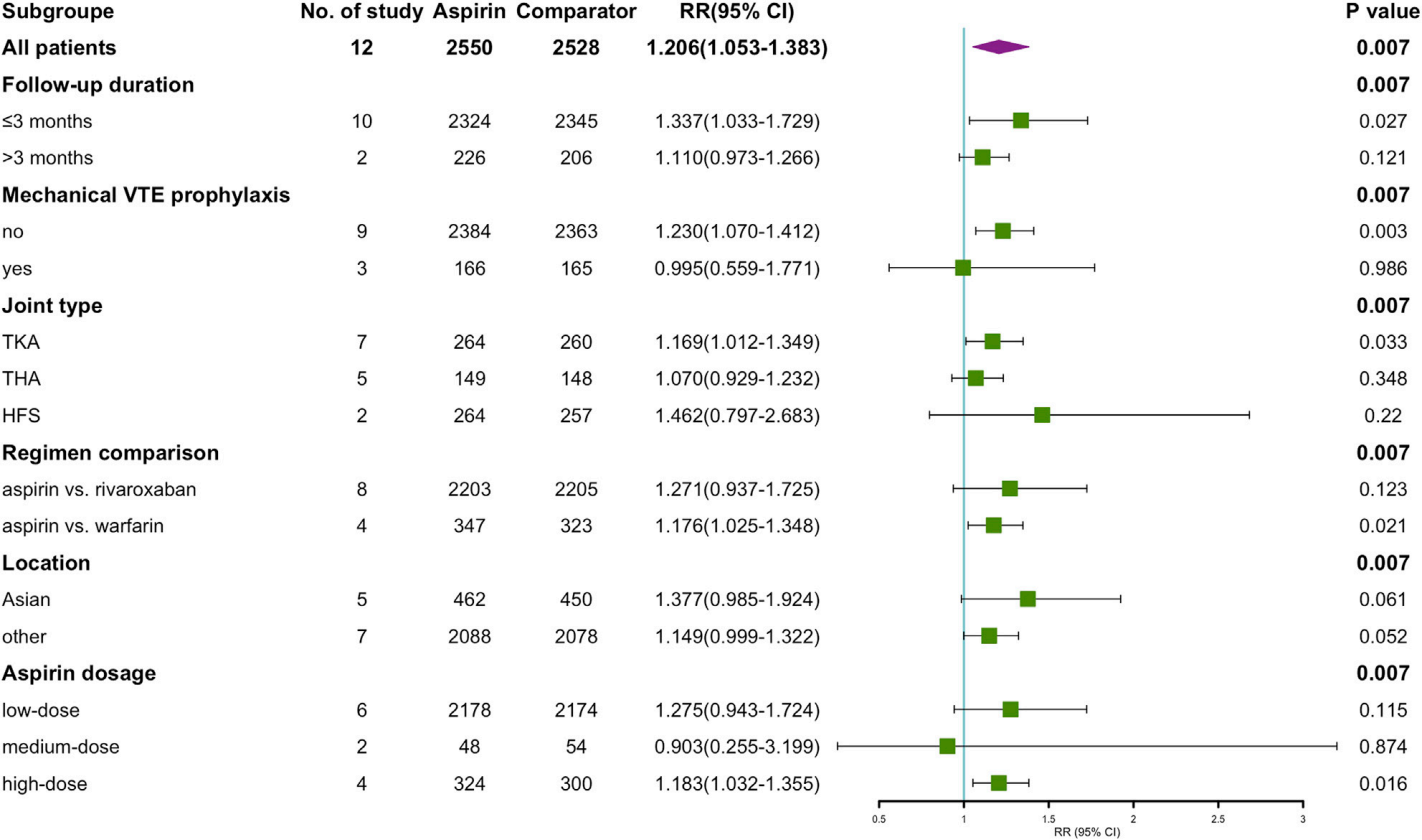
Summary of aspirin vs. OAC on VTE occurrence after subgrouping according to study-level characteristics.

To analyze our primary results more comprehensively, we assessed several potentially confounding variables for subgroup analyses, including the type of comparators (rivaroxaban or warfarin), duration of follow-up (within 3 months or more than 3 months), mechanical prophylaxis of thrombosis (yes or no), country in which the trial was conducted (Asia or non-Asia), type of joint type (TKA, THA or HFS) and aspirin dose (81–162 mg qd, 200–300 mg qd or 650 mg qd). In all pre-2000 studies, the oral anticoagulant (OAC) in the control group was warfarin. The results of the subgroup analysis are shown in Figure 8. There was no significant difference in RR of aspirin compared to OAC in Asia and non-Asia. While in other subgroups, the number of VTE events was higher in the aspirin group than in the control group. These included: follow-up ≤3 months (RR = 1.337, *p* = 0.027), no combined use of mechanical prophylaxis (RR = 1.230, *p* = 0.003), type of procedure as TKA (RR = 1.169, *p* = 0.033), control as warfarin (RR = 1.176, *p* = 0.021), and a daily dose of aspirin ≥650 mg (RR = 1.183, *p* = 0.016).

## 4 Discussion

VTE is the most common cause of perioperative hospital deaths, and its complications consume a significant amount of healthcare resources (Reddy et al., 2022). Anticoagulants have been shown to reduce postoperative mortality and VTE-related complications (Hu et al., 2021). Aspirin has the advantage of being widely available, inexpensive, does not require monitoring, and is traditionally considered to have a lower bleeding risk than anticoagulants in the perioperative period (Diep and Garcia, 2020). In the PEP study (O’Brien et al., 2000) 17,000 patients undergoing hip fracture surgery or elective arthroplasty were included were randomly assigned to 160 mg aspirin or placebo per day for 35 days starting preoperatively. Based on this evidence, several clinical guidelines (Falck-Ytter et al., 2012; Anderson et al., 2019; Gee, 2019) consider aspirin as one of the drug choices for thromboprophylaxis. The popularity of aspirin is increasing owing to the guideline recommendations. According to US statistics, oral anticoagulants and aspirin are the main part of anticoagulant prescriptions after hip arthroplasty, and aspirin prescriptions are on the rise year by year (Agarwal et al., 2023).

We included 12 studies evaluating the efficacy and safety of aspirin versus oral anticoagulants for the prevention of VTE after total joint arthroplasty or hip fracture surgery. The analysis of the primary outcome showed that a higher number of VTE occurred with aspirin compared with oral anticoagulants (RR = 1.206, 95% CI 1.053–1.383), this indicated that the clinical efficacy of OAC was superior to that of aspirin. This result is similar to that of a large observational cohort (Richardson et al., 2019). However, previous meta-analyses such as the study conducted by Matharu et al. (2020) and the study conducted by Singjie et al. (2022), comparing the efficacy and safety of aspirin and other anticoagulants, including oral anticoagulants and LMWH, in the prevention of VTE after hip and knee replacement or orthopedic trauma, have shown that there is no statistically significant difference in the prevention of VTE between aspirin and anticoagulants. These results differed from our findings. While it may be related to the different types of anticoagulants in the control group, to explain our findings further, we performed additional subgroup analyses based on the characteristics of the included studies.

First, we defined possible factors that would affect RR, including the medication regimen for thromboprophylaxis, such as aspirin dose, and type of comparator. Follow-up time, trial location, and type of surgery were also considered. To avoid duplicating analyses, the year of publication (pre-2000 vs. post-2000) was not considered. Moreover, current guidelines (Chinese Orthopaedic Association, 2010; Falck-Ytter et al., 2012; Anderson et al., 2019; Gee, 2019) do not standardize the recommended duration of anticoagulation. In addition, the duration of prophylaxis can be influenced by factors such as the type of procedure and individual VTE risk, making it difficult to identify a standard for grouping anticoagulation durations for our analyses (Jones and Al-Horani, 2023).

Given that our control group contained two anticoagulants, warfarin and rivaroxaban, analyzed separately according to the type of anticoagulant, it was found that aspirin and rivaroxaban showed similar effects in preventing venous thrombosis. This is consistent with the results of a large cohort observational study (Bala et al., 2017) and previous meta-analyses (Kapoor et al., 2017; Matharu et al., 2020; Hu et al., 2021). However, in comparison to warfarin, aspirin demonstrated a lower efficacy in preventing VTE. Interestingly, from a pharmacological perspective, aspirin’s antiplatelet effect is immediate, while warfarin’s anticoagulant effect is delayed by 2–3 days, and the response dose-response can be unstable due to patient-related or genetic factors because thrombosis is more likely to occur during the surgical period, the value of delayed anticoagulation may be lower (Della Valle et al., 2006). In a systematic review, warfarin was found to have a higher risk of symptomatic DVT compared to low-dose aspirin (Azboy et al., 2020). We believe that the RCT studies in the warfarin subgroup were all published before 2000, and that both of these indistinguishable factors, i.e., the year of publication and the type of anticoagulant, may have had an impact on the RR for the primary outcome; therefore, we are unable to confirm whether aspirin is not as effective as warfarin in preventing VTE in orthopedic surgery. Of note, the PEPPER trial was a large pragmatic clinical trial (NCT02810704) designed to assess the combined effect of three antithrombotic drugs (aspirin, warfarin, and rivaroxaban) on overall mortality and symptomatic VTE in patients receiving TKA and THA (Pellegrini, Jr et al., 2022). The results from this trial may give us new perspectives.

We also analyzed the results for other subgroups. The subgroups of the trial location did not have statistically significant results for RR (RR = 1.149, *p* = 0.052). However, aspirin was less effective as an anticoagulant than the control group in the remaining subgroups: follow-up within 3 months, use of high-dose aspirin (325 mg bid), no combination of mechanical thromboprophylaxis, and the type of procedure was TKA. Generally, VTE after major orthopedic surgery occurs more frequently within the first 3 months, the majority of symptomatic VTE (94%) occurs within 2 weeks after joint replacement surgery, with 89% occurring within the first week (Karasavvidis et al., 2022). This difference in risk helps explain the higher number of VTEs in the aspirin group at 3 months of follow-up. In terms of procedure type, the risk of venous thrombosis varies among the three major orthopedic procedures, with TKA having a higher propensity for thrombosis and a slightly higher incidence of VTE(Gionis et al., 2013). Considering that only two studies involving HFS were included in this meta-analysis, with a small sample size, and that in Huang’s study (Huang et al., 2019), the majority of patients were recruited postoperatively, further validation of the effectiveness of aspirin in patients with HFS is needed. Regarding aspirin dosage, recent studies have shown that low-dose aspirin (81 mg, bid) is comparable to high-dose aspirin (325 mg, bid) in preventing VTE after total joint replacement (0.6% vs. 1.3%, *p* = 0.62) (Shafiei et al., 2023). In the study by Merkow et al. (2021), patients with TKA had a higher incidence of VTE with high-dose aspirin (325 mg, bid) than in the low-dose aspirin group (81 mg, bid) (1.41% vs. 0.23%, *p* < 0.001). The results of these two studies and ours support the opinion that low-dose aspirin seems to be the more reasonable choice. Moreover, the subgroup analysis results suggest that, in comparison to utilizing aspirin alone, the combined use of mechanical thromboprophylaxis appears to confer clinical benefits, aligning with findings from a systematic review focusing on major orthopedic surgeries (Balk, M.D., M.P.H. et al., 2017). This review demonstrated that in TKA patients, the overall incidence of deep vein thrombosis in the combined mechanical device and antiplatelet group was lower than in the antiplatelet-only group. According to the American Society of Hematology (ASH) guidelines from 2019, combination prophylaxis or mechanical prophylaxis alone is recommended based on individual patient and surgical type VTE and bleeding risk (Anderson et al., 2019).

In terms of safety outcomes, there was no statistical difference between aspirin and OAC in major bleeding, mortality, or wound-related issues (*p* > 0.05). Also, aspirin was associated with fewer minor bleeding and fewer total bleeding events. In fact, aspirin has been traditionally recognized as having the lowest rate of bleeding complications in surgical patients (Pellegrini et al., 2020). Several large observational cohort studies have demonstrated that the risk of bleeding with postoperative aspirin use in patients with TKA or THA is negligible, making the prospect of aspirin use exciting (Reitman et al., 2003; Lotke and Lonner, 2006; Agaba et al., 2017).

## 5 Strengths and limitations

There have been many studies on the prevention of VTE with aspirin, including enoxaparin versus aspirin and aspirin versus placebo, and few studies have meta-analyzed aspirin with oral anticoagulants. This study collects research on aspirin versus oral anticoagulants and comprehensively analyzes the efficacy and safety of these two medications in the prevention of VTE after major orthopedic surgery. It provides reference recommendations for the safe use of aspirin for thrombosis prevention in the future. Second, this meta-analysis included 12 RCTs involving a large sample of 5,088 patients, six of which were published in the literature in the last 5 years, with reliable results. In addition, only RCTs were included in this study to avoid unreliable outcomes due to different trial design methods.

Of course, some limitations of this meta-analysis exist. First, although we searched for DOACs including rivaroxaban, dabigatran, and others, limited data prevented us from comparing the efficacy and safety of aspirin with DOACs other than rivaroxaban. Moreover, we performed subgroup analyses of the primary outcomes, but could not exclude all potential confounders in the baseline characteristics because data on individual patient clinical characteristics were not available. Thirdly, any of the factors influencing patient mortality in the included studies were not clearly described and therefore could not be completely avoided. Finally, due to the low incidence of outcomes, the classic half-integer correction was applied, which may have contributed to a slight overestimation of event rates.

## 6 Conclusion

Available evidence from the RCT suggests that aspirin is statistically inferior to oral anticoagulants regarding the clinical effectiveness of postoperative prophylactic anticoagulation after major orthopedic surgery. Further subgroup analysis revealed that aspirin was similar to rivaroxaban and inferior to warfarin. Considering that the confounding factor of year of publication could not be excluded, our results need to be further validated. In the safety events of all-cause mortality, major bleeding, and wound-related events, there were no statistically significant differences from oral anticoagulants. Aspirin remains promising for use in patients after major orthopedic surgery owing to its advantages in reducing the overall risk of bleeding.

## Data Availability

The original contributions presented in the study are included in the article/Supplementary Material, further inquiries can be directed to the corresponding authors.
