# Supplementary material for: Comparison of efficacy and safety between aspirin and oral anticoagulants for venous thromboembolism prophylaxis after major orthopaedic surgery: a meta-analysis of randomized clinical trials
Source: Front Pharmacol. 2024 Jan 8;14:1326224. doi: 10.3389/fphar.2023.1326224 (PMC10800895; doi:10.3389/fphar.2023.1326224)
Supplement: Supplementary file 1 [file DataSheet1.zip › Supplementary Material/Supplementary Tables 2-9.docx]

**Supplementary Material**

**Supplementary Table 2. Results of the egger test for meta-analysis.**

| **Outcome** | **No. of included studies** | **P value** |
| --- | --- | --- |
| VTE occurrence | 12 | 0.345 |
| All-Cause Mortality | 11 | 0.035 |
| Major bleeding | 10 | 0.652 |
| Minor bleeding | 10 | 0.853 |
| Total bleeding | 10 | 0.970 |
| Wound-related issues | 8 | 0.673 |

**Supplementary Table 3. Sensitivity analysis of studies included in the analysis of VTE occurrence.**

| **Study omitted** | **Estimate** | **95%CI** |
| --- | --- | --- |
| Huang et al, 2019 | 0.230 | -0.034 - 0.495 |
| Hongnaparak et al, 2022 | 0.197 | -0.025 - 0.420 |
| Anderson et al, 2017 | 0.215 | -0.014 - 0.446 |
| Jiang et al, 2014 | 0.218 | -0.012-0.448 |
| Lotke et al, 1996 | 0.274 | -0.013 - 0.561 |
| Powers et al, 1989 | 0.094 | -0.021 - 0.210 |
| Ren et al, 2021 | 0.200 | -0.025 - 0.426 |
| Salzman et al, 1971 | 0.206 | -0.022 - 0.435 |
| Woolson et al, 1991 | 0.202 | -0.028 - 0.432 |
| Zou et al, 2014 | 0.105 | -0.009 - 0.219 |
| Colleoni et al, 2018 | 0.199 | -0.019 - 0.419 |
| Zhou et al, 2023 | 0.179 | -0.038 - 0.396 |

95%CI: 95% confidence interval

**Supplementary Table 4. Sensitivity analysis of studies included in the analysis of mortality.**

| **Study omitted** | **Estimate** | **95%CI** |
| --- | --- | --- |
| Huang et al, 2019 | 0.211 | -0.783 - 1.205 |
| Hongnaparak et al, 2022 | 0.209 | -0.785 - 1.204 |
| Anderson et al, 2017 | 0.152 | -0.852 - 1.157 |
| Jiang et al, 2014 | 0.209 | -0.785 - 1.204 |
| Powers et al, 1989 | -0.046 | -1.229 - 1.135 |
| Ren et al, 2021 | 0.205 | -0.788 - 1.200 |
| Salzman et al, 1971 | 0.209 | -0.785 - 1.204 |
| Woolson et al, 1991 | 0.212 | -0.782 - 1.206 |
| Zou et al, 2014 | 0.214 | -0.780 - 1.208 |
| Colleoni et al, 2018 | 0.307 | -0.705 - 1.320 |
| Zhou et al, 2023 | 0.209 | -0.785 - 1.204 |

95%CI: 95% confidence interval

**Supplementary Table 5**. **Sensitivity analysis of studies included in the analysis of major bleeding events.**

| **Study omitted** | **Estimate** | **95%CI** |
| --- | --- | --- |
| Huang et al, 2019 | -0.052 | -0.764 - 0.659 |
| Hongnaparak et al, 2022 | 0.008 | -0.684 - 0.701 |
| Anderson et al, 2017 | -0.268 | -1.130 - 0.594 |
| Powers et al, 1989 | 0.194 | -0.525 - 0.915 |
| Ren et al, 2021 | 0.008 | -0.684 - 0.701 |
| Salzman et al, 1971 | 0.094 | -0.681 - 0.869 |
| Woolson et al, 1991 | 0.008 | -0.684 - 0.700 |
| Zou et al, 2014 | 0.008 | -0.684 - 0.700 |
| Colleoni et al, 2018 | 0.000 | -0.692 - 0.693 |
| Zhou et al, 2023 | 0.008 | -0.695 - 0.712 |

95%CI: 95% confidence interval

**Supplementary Table 6**. **Sensitivity analysis of studies included in the analysis of minor bleeding events.**

| **Study omitted** | **Estimate** | **95%CI** |
| --- | --- | --- |
| Huang et al, 2019 | -0.376 | -0.579 - -0.172 |
| Hongnaparak et al, 2022 | -0.376 | -0.580 - -0.172 |
| Anderson et al, 2017 | -0.419 | -0.628 - -0.210 |
| Powers et al, 1989 | -0.387 | -0.589 - -0.184 |
| Ren et al, 2021 | -0.373 | -0.576 - -0.171 |
| Salzman et al, 1971 | -0.388 | -0.592 - -0.184 |
| Woolson et al, 1991 | -0.380 | -0.582 - -0.178 |
| Zou et al, 2014 | -0.333 | -0.789 - 0.123 |
| Colleoni et al, 2018 | -0.381 | -0.582 - -0.179 |
| Zhou et al, 2023 | -0.342 | -0.548 - -0.137 |

95%CI: 95% confidence interval

**Supplementary Table 7. Sensitivity analysis of studies included in the analysis of total bleeding events.**

| **Study omitted** | **Estimate** | **95%CI** |
| --- | --- | --- |
| Huang et al, 2019 | -0.331 | -0.584 - -0.079 |
| Hongnaparak et al, 2022 | -0.320 | -0.570 - -0.070 |
| Anderson et al, 2017 | -0.408 | -0.610 - -0.205 |
| Powers et al, 1989 | -0.321 | -0.552 - -0.091 |
| Ren et al, 2021 | -0.329 | -0.548 - -0.110 |
| Salzman et al, 1971 | -0.367 | -0.564 - -0.170 |
| Woolson et al, 1991 | -0.328 | -0.574 - -0.082 |
| Zou et al, 2014 | -0.219 | -0.595 - 0.156 |
| Colleoni et al, 2018 | -0.330 | -0.572 - -0.089 |
| Zhou et al, 2023 | -0.309 | -0.506 - -0.112 |

95%CI: 95% confidence interval

**Supplementary Table 8**. **Sensitivity analysis of studies included in the analysis of Wound-related issues.**

| **Study omitted** | **Estimate** | **95%CI** |
| --- | --- | --- |
| Huang et al, 2019 | -0.545 | -1.231 - 0.139 |
| Hongnaparak et al, 2022 | -0.407 | -1.064 - 0.249 |
| Jiang et al, 2014 | -0.447 | -1.100 - 0.204 |
| Lotke et al, 1996 | -0.562 | -1.340 - 0.216 |
| Ren et al, 2021 | -0.494 | -1.140 - 0.152 |
| Woolson et al, 1991 | -0.488 | -1.134 - 0.157 |
| Zou et al, 2014 | -0.371 | -1.053 - 0.311 |
| Colleoni et al, 2018 | -0.429 | -1.087 - 0.228 |

95%CI: 95% confidence interval

**Supplementary Table 9. The summary of the results of the meta-analysis for the outcomes.**

| **Outcome** | **No. of included studies** | **RR** | **95% CI** | **P value** |
| --- | --- | --- | --- | --- |
| VTE occurrence | 12 | 1.206 | 1.053-1.383 | 0.007 |
| All-Cause Mortality | 11 | 1.208 | 0.459-3.177 | 0.702 |
| Major bleeding | 10 | 0.952 | 0.499-1.815 | 0.882 |
| Minor bleeding | 10 | 0.685 | 0.552-0.850 | 0.001 |
| Total bleeding | 10 | 0.726 | 0.590-0.892 | 0.002 |
| Wound-related issues | 8 | 0.618 | 0.333-1.145 | 0.126 |

RR: relative risk; 95%CI: 95% confidence interval
